# Neutrophil Survival Signaling During *Francisella tularensis* Infection

**DOI:** 10.3389/fcimb.2022.889290

**Published:** 2022-07-06

**Authors:** Lauren C. Kinkead, Samantha J. Krysa, Lee-Ann H. Allen

**Affiliations:** ^1^Inflammation Program, University of Iowa, Iowa City, IA, United States; ^2^Department of Microbiology and Immunology, University of Iowa, Iowa City, IA, United States; ^3^Iowa City VA Health Care System, Iowa City, IA, United States; ^4^Molecular Medicine Training Program, University of Iowa, Iowa City, IA, United States; ^5^Department of Medicine, Division of Infectious Diseases, University of Iowa, Iowa City, IA, United States; ^6^Harry S. Truman Memorial VA Hospital, Columbia, MO, United States; ^7^Department of Molecular Microbiology and Immunology, University of Missouri, Columbia, MO, United States

**Keywords:** neutrophils, apoptosis, PI 3-kinase, P38 MAP kinase, *Francisella tularensis*

## Abstract

Neutrophils are the most abundant and shortest-lived leukocytes in humans and tight regulation of neutrophil turnover *via* constitutive apoptosis is essential for control of infection and resolution of inflammation. Accordingly, aberrant neutrophil turnover is hallmark of many disease states. We have shown in previous work that the intracellular bacterial pathogen *Francisella tularensis* markedly prolongs human neutrophil lifespan. This is achieved, in part, by changes in neutrophil gene expression. Still unknown is the contribution of major neutrophil pro-survival signaling cascades to this process. The objective of this study was to interrogate the contributions of ERK and p38 MAP kinase, Class I phosphoinositide 3-kinases (PI3K), AKT, and NF-κB to neutrophil survival in our system. We demonstrate that both ERK2 and p38α were activated in *F. tularensis*-infected neutrophils, but only p38α MAPK was required for delayed apoptosis and the rate of cell death in the absence of infection was unchanged. Apoptosis of both infected and uninfected neutrophils was markedly accelerated by the pan-PI3K inhibitor LY2094002, but AKT phosphorylation was not induced, and neutrophil death was not enhanced by AKT inhibitors. In addition, isoform specific and selective inhibitors revealed a unique role for PI3Kα in neutrophil survival after infection, whereas only simultaneous inhibition of PI3Kα and PI3kδ accelerated death of the uninfected controls. Finally, we show that inhibition of NF-κB triggered rapid death of neutrophil after infection. Thus, we defined roles for p38α, PI3Kα and NF-κB delayed apoptosis of *F. tularensis*-infected cells and advanced understanding of Class IA PI3K isoform activity in human neutrophil survival.

## Introduction

Polymorphonuclear leukocytes (PMNs, neutrophils) are critical innate immune cells that are among the first cells recruited to a site of an infection. These cells make up the majority of the white blood cells in circulation and are turned over on the order of 10^11^ cells per day (Athens et al., 1961). Notably, these phagocytes are essential for innate host defense and utilize a combination of reactive oxygen species (ROS), antimicrobial peptides, and proteolytic enzymes to kill invading microorganisms (Nauseef, 2007a).

Neutrophils have an intrinsically short lifespan of less than 24 hours in the bloodstream and undergo constitutive apoptosis, an active, tightly regulated form of cell death that is essential for homeostasis (Akgul et al., 2001; Luo and Loison, 2008; McCracken and Allen, 2014). Various stimuli can alter the rate of constitutive apoptosis including cytokines, microbial molecular patterns and phagocytosis (Kennedy and DeLeo, 2009; McCracken and Allen, 2014). In particular, phagocytosis and ROS production typically accelerate neutrophil cell death and lead to efficient engulfment of dying cells by macrophages (Rigby and DeLeo, 2012). Not only does this process aid in pathogen elimination, it also reprograms macrophages to a pro-resolving, anti-inflammatory phenotype that is essential for resolution of the inflammatory response (Kennedy and DeLeo, 2009; Rigby and DeLeo, 2012). Accordingly, dysregulation of this process sustains infection and exacerbates host tissue destruction and exemplifies a host response that is both aberrant and ineffective (Kennedy and DeLeo, 2009; McCracken and Allen, 2014).

Neutrophil apoptosis occurs when the levels of numerous proapoptotic factors outweigh the levels of antiapoptotic factors in the cell. Constitutive apoptosis is governed by the intrinsic pathway, and at the core of this pathway are mitochondria. Outer mitochondrial membrane (OMM) permeabilization is one of the early events in the intrinsic apoptotic pathway and is mediated by two major proapoptotic factors of the BCL-2 family, BAX and BAK (McCracken and Allen, 2014). These proteins oligomerize and insert into the OMM, thus disrupting membrane potential and mediating release of inner membrane space (IMS) proteins cytochrome *c*, SMAC and HTRA2. which initiate apoptosis *via* caspase-9 and the apoptosome (Schwartz et al., 2013; McCracken and Allen, 2014). Antiapoptotic factors such as MCL-1 and A1 prevent OMM disruption and are present in excess in healthy neutrophils but diminish in abundance as cells age (Edwards et al., 2004; McCracken and Allen, 2014). Additional regulation is mediated by cellular inhibition of apoptosis proteins such XIAP, which acts downstream of mitochondria and inhibits caspase-9 and caspase-3 by direct binding (McCracken and Allen, 2014).

To avert apoptosis, neutrophils must receive one or more survival cues that are robust enough to sustain an excess of pro-survival/antiapoptotic regulators that control cell lifespan. The major pro-survival pathways in neutrophils are overlapping and interconnected and include signaling cascades mediated by phosphoinositide 3-kinase (PI3K)/AKT, MEK/ERK and p38 MAP kinases (MAPKs) and NF-κB which are differentially activated by growth factors, ligation of β2 integrins, inflammatory mediators such as IL-8 and C5a and microbial products and pathogens (Luo and Loison, 2008; Geering and Simon, 2011; McCracken and Allen, 2014).

Several pathogenic microbes have evolved strategies to circumvent neutrophil antimicrobial mechanisms and promote neutrophil survival to maintain a site for replication and immune evasion inside the host (DeLeo, 2004; McCracken and Allen, 2014). This group of pathogens includes, but is not restricted to, *Anaplasma phagocytophilum*, *Chlamydia pneumoniae*, *Leishmania major* and *Coxiella burnetii*. Each of these microbes utilizes a specific mechanism of apoptosis inhibition that involves manipulation of anti-apoptosis regulators and one or more of the survival signaling cascades (Kim and Rikihisa, 2002; Lee and Goodman, 2006; Du et al., 2011; McCracken and Allen, 2014; Sarkar et al., 2015; Cherla et al., 2018).

*Francisella tularensis* is a Gram-negative, facultative intracellular bacterium and the causative agent of the zoonosis tularemia. *F. tularensis*, subspecies *tularensis* (type A), and subspecies *holarctica* (type B) differ in geographic distribution and account for nearly all human infections with this organism (McLendon et al., 2006). Inhalation of as few as ten bacteria can result in a severe bronchopneumonia that is coupled to bacterial dissemination to the liver, spleen and other organs and has a mortality rate of approximately 30-60% if untreated (Dennis et al., 2001). *F. tularensis* utilizes a variety of strategies to modulate the innate immune response and its effectors and infects several cell types, including macrophages and neutrophils (Kinkead and Allen, 2016). In all infected cell types, *F. tularensis* uses its type VI secretion system to escape the phagosome and replicate in the cytosol (McCaffrey and Allen, 2006; Kinkead and Allen, 2016). Prior to phagosome escape, FevR and other virulence factors allow evasion of toxic oxidants by inhibiting NADPH oxidase assembly and activity, whereas an atypical LPS and O-antigen capsule prevent bacterial detection by TLR4 and confer resistance to complement-mediated lysis, respectively (McCaffrey et al., 2010; Kinkead and Allen, 2016).

Importantly, studies in animal models revealed that macrophages and neutrophils play distinct roles in tularemia pathogenesis, such that macrophages are major vehicles for bacterial dissemination whereas neutrophils mediate destruction of infected tissues (Kinkead and Allen, 2016). Thus, when bacteria are inhaled, alveolar macrophages are infected first, but recruited neutrophils soon outnumber macrophages and are the dominant leukocyte present throughout acute infection. From day two onward, infected neutrophils, bacteria and necrotic debris progressively clog alveolar spaces, whereas macrophages disseminate infection to distal organs. Neutrophilia correlates with disease severity and tissue destruction, and blocking PMN migration into the lungs allows mice to survive an otherwise lethal infection (Malik et al., 2007; Kinkead and Allen, 2016).

As defects in PMN turnover exacerbate their destructive potential, we hypothesized that *F. tularensis* may manipulate neutrophil death as part of its virulence strategy and went on to demonstrate that both type A and type B *F. tularensis* strains modulate the major apoptotic pathways in neutrophils to significantly delay apoptosis (Schwartz et al., 2012; Schwartz et al., 2013; McCracken et al., 2016). This delay involves inhibition of caspase processing and activation and sustained mitochondrial membrane integrity that coincides with impaired BAX translocation, and increased expression of genes encoding antiapoptotic factors such as XIAP, A20, FLIP, cIAP2 and calpastatin (Schwartz et al., 2012; Schwartz et al., 2013; McCracken et al., 2016).

Despite prior studies, the molecular mechanisms of *F. tularensis*-mediated neutrophil apoptosis inhibition are incompletely defined. The objective of this study was to elucidate the pro-survival signaling pathways that are required to extend neutrophil lifespan during this infection. As NF-κB controls expression of many anti-apoptosis factor genes, we hypothesized that activity of this transcription factor would be essential. On the other hand, we also hypothesized that pathways linked to growth factor signaling and IL-8 (CXCL8) would be dispensable, as our studies utilize a serum-free infection model and because *F. tularensis*-infection does not elicit PMN IL-8 secretion (Schwartz et al., 2012). Herein, we demonstrate that survival of *F. tularensis*-infected human neutrophils is specifically dependent on the activity of NF-κB, PI3Kα and p38 MAPK, but not MEK/ERK, AKT or other Class I PI3K isoforms.

## Materials and Methods

### Isolation of Human Neutrophils

Heparinized venous blood was obtained from healthy adult volunteers with no history of tularemia in accordance with protocols approved by the Institutional Review Board for Human Subjects at the University of Iowa (201609850 and 200307026) and the University of Missouri (20331322). Neutrophils were isolated using a sequential dextran sedimentation, density-gradient separation with Ficoll-Hypaque (GE Healthcare, Little Chalfont, UK) and hypotonic erythrocyte lysis as previously described (Nauseef, 2007b). Using this method, purity was >95% PMNs. PMNs were suspended in Hank’s balanced salt solution (HBSS) without divalent cations (Fisher Scientific, Hampton, NH) enumerated, and diluted to 2x10^7^/ml. In all cases, replicate experiments were performed using PMNs from different donors.

### Bacterial Strains and Growth Conditions

*F. tularensis* subspecies *holarctica* live vaccine strain (LVS) has been previously described and retains key features of fully virulent *F. tularensis* in human neutrophils *in vitro* but requires only BSL-2 containment (McCaffrey et al., 2010; Schwartz et al., 2012; Schwartz et al., 2013). Bacteria were inoculated onto Difco cysteine heart agar (BD Biosciences, San Jose, CA) supplemented with 9% defibrinated sheep blood (Remel, Lenexa, KS) (CHAB) and grown for 48 hours at 37°C with humidity in 5% CO_2_. Unless otherwise stated, cultures of LVS were started at an OD_600_ of 0.01 in Bacto brain heart infusion (BD Biosciences) (BHI) broth and incubated overnight (14-18 hours), shaking at 200 rpm. Overnight cultures were diluted again and incubated at 37°C in 5% CO_2_, shaking at 200 rpm, for 2-4 hours. Mid-exponential growth phase bacteria were harvested and washed once with HBSS containing divalent cations (Fisher Scientific).

### Infection and Culture of Neutrophils

Washed bacteria were quantified by measurement of absorbance at 600 nm. Unless otherwise stated, PMNs (5x10^6^/ml) were diluted in HEPES-buffered RPMI-1640 containing L-glutamine and phenol red (Lonza, Walkersville, MD) in the absence of serum and infected with LVS to achieve 20 bacteria/cell as previously described (McCracken et al., 2016). Cultures (1-2 ml each) were incubated in 14 ml polypropylene snap-cap tubes at 37°C with humidity and 5% CO_2_ for up to 24 hours.

### Phospho-MAPK Dot Blots

The Proteome Profiler™ human phospho-MAPK slide array kits (R&D Systems Inc., Minneapolis, MN) were used for detection of 24 different MAPK proteins in PMNs. Whole-cell lysates were prepared according to the manufacturer’s instructions. Briefly, neutrophils were left untreated or were infected with LVS, and 2, 10 or 24 hours later neutrophils were pelleted by centrifugation at 250 g. Cell pellets were resuspended in lysis buffer containing aprotinin, leupeptin, PMSF, AEBSF, levamisole, bestatin, E-64, and pepstatin A (Sigma-Aldrich, St. Louis, MO), supplemented with the Pierce Halt™ phosphatase inhibitor cocktail (sodium fluoride, sodium orthovanadate, sodium pyrophosphate and β-glycerophosphate) (Thermo Fisher Scientific, Waltham, MA). Protein concentrations were determined by performing a Pierce bicinchoninic acid (BCA) protein assay (Thermo Fisher Scientific), according to the manufacturer’s instructions. Array dot blots were processed according to the manufacturer’s protocol.

### Assessment of Neutrophil Apoptosis

Neutrophil apoptosis was measured by flow cytometric analysis of phosphatidylserine (PS) externalization using an Accuri C6 flow cytometer (BD Biosciences, Franklin Lakes, NJ). PMNs (5x10^5^) were co-stained with Annexin V-FITC (BioVision, Milpitas, CA) and propidium iodide (PI) (BioVision) in binding buffer (10 mM HEPES pH 7.4, 140 mM NaCl, 2.5mM CaCl_2_) according to the manufacturer’s instructions. For each sample, approximately ten thousand events were collected, and the data were analyzed using cFlow software (BD Biosciences). Unless otherwise stated, graphs include Annexin V-positive/PI-negative (early) and Annexin V/PI double-positive (late apoptotic) cells.

Progression to apoptosis was also assessed independently using established methods to quantify nuclear condensation (Schwartz et al., 2012; McCracken et al., 2016; Kinkead et al., 2018). In brief, after incubation at 37°C for 18 hours, control, infected and/or drug-treated neutrophils were attached to glass coverslips by cytocentrifugation and then fixed and stained using with Hema-3 reagents (Fisher Scientific). Coverslips were attached to slides using Permount (Fisher Scientific) and analyzed using a Zeiss Axioplan 2 light microscope (Carl Zeiss Inc., Thornwood, NY). Neutrophils in random fields of view were scored as having healthy nuclei comprised of 3-4 interconnected lobes, or apoptotic, condensed (spherical) nuclei and at least 100 cells/coverslip/sample were assessed (Schwartz et al., 2012; McCracken et al., 2016; Kinkead et al., 2018).

### Inhibition of Signaling Pathways

All inhibitors were resuspended from powder in tissue culture-grade dimethyl sulfoxide (DMSO) (Sigma-Aldrich). Neutrophils were pretreated with various inhibitors of survival signaling (**Table 1**) at 37°C with 5% CO_2_ for 30-60 min, as indicated, after which *F. tularensis* LVS was added to corresponding cultures. Neutrophil apoptosis was quantified after further incubation at 37°C for 4-24 hours, as indicated, using flow cytometry after Annexin V-FITC/PI staining and analysis of nuclear morphology as described above.

**Table 1:** Inhibitors.

Inhibitor	Target	[Final]	Pre-treatment	Supplier
Afuresertib (GSK2110183)	AKT1/2/3	10 μM	60 min	SelleckChem, Houston, TX
CAPE	NF-κB	100 μM	60 min	Calbiochem, San Diego, CA
GDC-0941 Pictilisib	PI3Kα,δ	20 μM	60 min	Cayman Chemical, Ann Arbor, MI
HS-173	PI3Kα	10 μM	60 min	SelleckChem
IC-87114	PI3Kβ, γ, δ*	1 μM	60 min	SelleckChem
LY294002	Pan PI3K	50 μM	30 min	Cell Signaling Technology, Danvers, MA
MK-2206	AKT1/2/3	1 μM	30 min	SelleckChem
NF-κB AI	NF-κB	30 μM	60 min	Calbiochem
PD98059	MEK1/2	50 μM	30 min	Calbiochem
PIK-294	PI3Kβ, γ, δ*	1 μM	60 min	Calbiochem
SB202190	p38 MAPK	30 μM	60 min	SelleckChem
SB203580	p38 MAPK	30 μM	60 min	Calbiochem
SB202474	SB03580 negative control	30 μM	30 min	Calbiochem
TGX-221	PI3Kβ*, γ, δ	100 μM	30 min	Cayman Chemical

*preferentially inhibited.

To account for possible detrimental effects of drug pretreatment, additional experiments were performed adding inhibitors simultaneously with or shortly after initiation of infection. In addition, to elucidate possible effects of each inhibitor on bacterial viability and fitness, drugs were added to BHI broth and LVS growth was quantified over 18 hours at 37°C by measurement of the OD_600_ (as described above).

As our published data demonstrate that both intracellular and extracellular LVS contribute to apoptosis inhibition (Schwartz et al., 2012; Kinkead et al., 2018) we sought to determine if phagocytosis influenced the effects of signaling inhibitors on apoptosis in our system. To this end, we prevented direct contact between neutrophils and bacteria using 0.4 μm Transwell inserts (Fisher Scientific) as we previously described (Schwartz et al., 2012). After incubation at 37°C for 24 hours, cells were processed for analysis of apoptosis by flow cytometry as described above.

### Detection of Phosphorylated, Intracellular Proteins *via* Flow Cytometry

Neutrophils were left untreated or infected with LVS as described above. At the indicated time points, PMNs were fixed in IC fixation buffer (eBioscience, Waltham, MA) for 30 minutes at room temperature. Following fixation, cells were pelleted by centrifugation at 250 g and permeabilized in 100% ice-cold methanol for 30 minutes on ice. After two washes in blocking buffer [Dulbecco’s phosphate-buffered saline (DPBS) without divalent cations (Fisher Scientific) supplemented with 0.5% bovine serum albumin (Sigma-Aldrich)], neutrophils were resuspended in blocking buffer at a concentration of 10^7^ cells/ml. Aliquots of neutrophils (10^6^) were mixed with purified human Fc binding inhibitor and anti-phospho-p38 MAPK (T180/Y182)-APC (clone 4NIT4KK, #17-9078-42) (from eBioscience). After incubation for 30 minutes at room temperature, stained cells were washed twice in blocking buffer and resuspended in DPBS without divalent cations for analysis on an Accuri C6 flow cytometer. Alternatively, cells control and infected neutrophils, or cells stimulated with 1 μM fMLF (Sigma-Aldrich) for 3 minutes in the presence and absence of 100 μM LY294002 (Cell Signaling Technology, Danvers, MA) were fixed and stained to detect active AKT using mouse anti-human phospho-AKT (S473)-APC (clone SDRNR, #17-9715) prior to analysis.

### Immunoblotting

Neutrophils were collected by centrifugation, washed, and then lysed in buffer containing 1% NP-40 along with the following protease and phosphatase inhibitors: aprotinin, leupeptin, AEBSF, PMSF, levimasole, bestatin, E-64, pepstatin A, diisopropylfluorophosphate, vanadate and sodium fluoride (McCracken et al., 2016). Alternatively, washed neutrophils were resuspended in relaxation buffer containing the protease and phosphatase inhibitors listed above and disrupted by nitrogen cavitation followed by separation of nuclei, cytosol and membrane fractions by differential centrifugation (Allen et al., 2005). Protein concentrations of each sample were determined using Pierce BCA assay kits. After boiling in sample buffer, proteins in each sample (5-10 μg) were separated on 4-12% NuPAGE Bis-Tris gradient gels and then transferred to polyvinylidene fluoride membranes by electroblotting. Blocked membranes were probed to detect: phospho-p38 MAPK (T180/Y182) using rabbit polyclonal Ab (pAb) #9211 (Cell Signaling Technology) at 1:500; AKT and phospho-AKT(S473) using rabbit pAb #9272 and #9271, respectively, (Cell Signaling Technology) each at 1:500; p110α using rabbit mAb clone C73F8 #4249T (Cell Signaling Technology) at 1:500; NF-κB p65 using clone D14E12 XP rabbit mAb, #8242 (Cell Signaling Technology) at 1:1,000; IκBα using rabbit mAb clone 44D4, #4812 (Cell Signaling Technology) at 1:1000; phospho-IκBα (S32) rabbit mAb clone 14D4, #2859 (Cell Signaling Technology) at 1:500; and β-actin using rabbit pAb #NB600-503SS (Novus Biologicals, Littleton, CO) at 1:2,000. In each case, bands were detected using horseradish peroxidase-conjugated secondary antibodies at 1:2,000 (Cytiva, Marlborough, MA) and Pierce SuperSignal West Femto chemiluminescence substrate (Thermo Fisher Scientific) followed by Li-COR Odyssey imaging (LI-COR Biosystems, Lincoln, NE).

### Statistical Analyses

Data are presented as the mean ± standard error of the mean (SEM) and were analyzed by two-way ANOVA followed by Tukey’s multiple comparisons post-tests. All analyses were performed using Prism version 7or 8 software (GraphPad, San Diego, CA). *p*-values less than 0.05 were considered statistically significant.

## Results

### p38 MAPK Is Activated and Required for Prolonged Survival of *F. tularensis*-Infected Neutrophils

**Figure 1:**
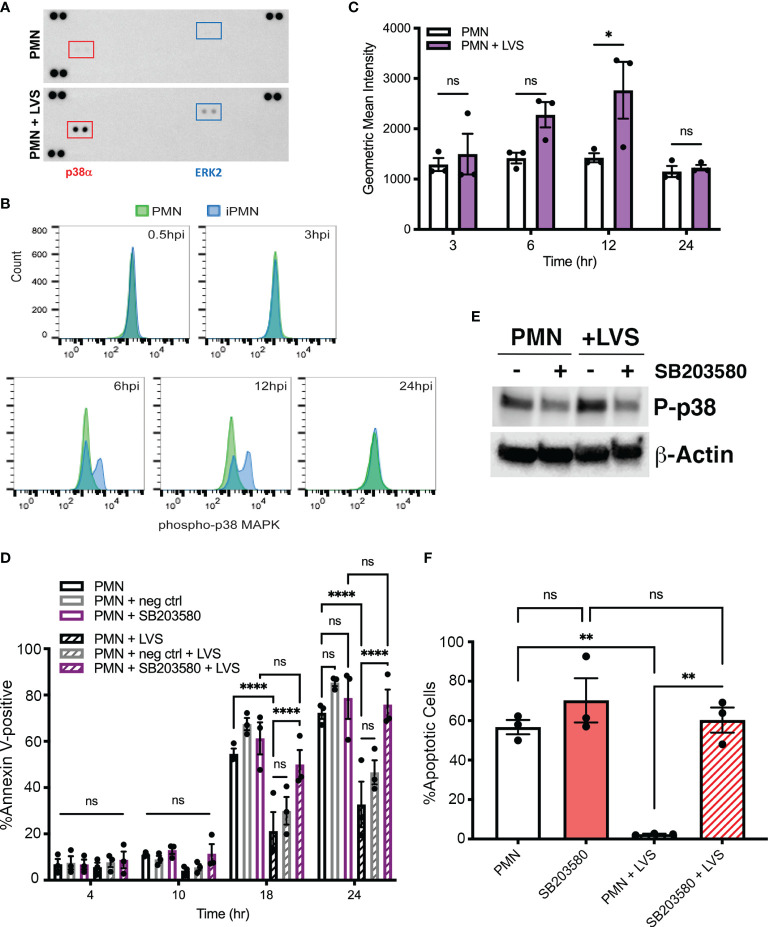
Infected neutrophil survival requires p38 MAPK activity. **(A)** Dot blot arrays show increased phosphorylation of p38α and ERK2 10 hours after LVS infection. Corner spots are positive and negative controls. Data shown are representative of three independent determinations. **(B, C)** Kinetics of p38 phosphorylation determined by intracellular straining and flow cytometry. Control cell data are in green and LVS-infected cell data (iPMN) are shown in blue. Representative histograms are shown in **(B)** and geometric mean intensities from three experiments are shown in **(C)**. ns, not significant. *p<0.05. **(D)** Effects of the p38 MAPK inhibitor SB203580 and its negative control SB202474 on apoptosis of control and LVS-infected neutrophils was quantified using Annexin V-FITC/PI staining and flow cytometry at 4, 10, 18 and 24 hours. Data are the mean ± SEM of three independent experiments. ns, not significant. ****p<0.0001, as indicated. **(E)** Immunoblots of of control and infected cell lysates prepared at 10 hours were probed to detect phosphorylated p38 with β-actin as the loading control. Data shown are representative of three determinations. **(F)** Percentage of neutrophils with condensed, apoptotic nuclei was quantified at 18 hours. Data are the mean ± SEM, n=3. Ns, not significant. **p<0.01.

MAPK signaling can be triggered by many stimuli that engage distinct cell surface receptors, including LPS and other TLR agonists, growth factors, and cytokines (Akgul et al., 2001). MEK/ERK MAPK and p38 MAPK pathways can act separately or together to modulate neutrophil apoptosis in response to specific stimuli (Klein et al., 2000; Klein et al., 2001; Geering and Simon, 2011; Tsai et al., 2013; McCracken and Allen, 2014; Cherla et al., 2018). To elucidate possible effects of *F. tularensis* on activation of MAPK signaling, we initially utilized phospho-MAPK dot blot arrays to survey intermediates in several MAPK cascades. To this end, dot blots were probed with lysates prepared from control neutrophils and their LVS-infected counterparts after 10 hours at 37°C. These data show that infection triggered increased phosphorylation of p38α MAPK and to a lesser extent ERK2 (**Figure 1A**). The dot blot spot map is shown in **Supplementary Figure 1A**.

To define the kinetics of p38 MAPK activation we utilized intracellular antigen staining and flow cytometry. (**Figures 1B, C**). By this assay, phosphorylation and activation of p38 MAPK was low or absent in early infection, peaked at 12 hours post-infection (hpi), and returned to baseline by 24 hpi. Dot blots of lysates prepared at 2 and 20 hours, together with the data in **Figure 1A**, confirmed this time course (**Supplementary Figure 1B**).

To examine the possible significance of MAPKs in neutrophil apoptosis inhibition by *F. tularensis* we utilized the p38 MAPK inhibitor SB203580 and included the related compound SB202474 as a negative control (Tsai et al., 2013). Specifically, neutrophils were left in medium alone or pretreated with 30 μM inhibitor prior to infection with LVS, and apoptosis was quantified 4, 10, 18 and 24 hours later using Annexin V-FITC/PI staining and flow cytometry as we described (McCracken et al., 2016; Kinkead et al., 2018). In all cases, uninfected/untreated cells were included as an additional control. Our data show that inhibition of p38 MAPK markedly accelerated apoptosis of infected PMNs by 18 hpi and completely eliminated the ability of LVS to extend neutrophil lifespan by 24 hpi, whereas the rate of apoptosis of uninfected neutrophils was unchanged (**Figures 1D**). Inhibitor efficacy was demonstrated by reduced phosphorylation of p38 detected by immunoblotting of cell lysates (**Figure 1E**). Specificity for p38 MAPK was indicated the lack of effect of the negative control compound, SB202474 (**Figure 1D**). DMSO, the diluent/vehicle control for all drugs used in this study, was also without effect (**Supplementary Figure 2**). Moreover, it is standard practice to pretreat neutrophils with inhibitors prior to stimulation or infection, and in this study PMNs were incubated with drugs for 30 or 60 min prior to addition of bacteria (**Table 1**). However, pretreatment was not essential, as similar results were obtained when the pretreatment step was omitted, and drugs were added simultaneously with initiation of infection or shortly thereafter (data not shown).

The nucleus of healthy human PMNs is comprised of 3-4 interconnected lobes that coalesce and condense into a sphere during apoptosis (Kennedy and DeLeo, 2009; Schwartz et al., 2012). This significant morphological change is a hallmark of apoptotic neutrophils and is readily apparent by light microscopy. Analysis of nuclear morphology at 18 hours confirmed the significant role of p38 MAPK in longevity of LVS-infected PMNs (**Figure 1F**).

**Figure 2:**
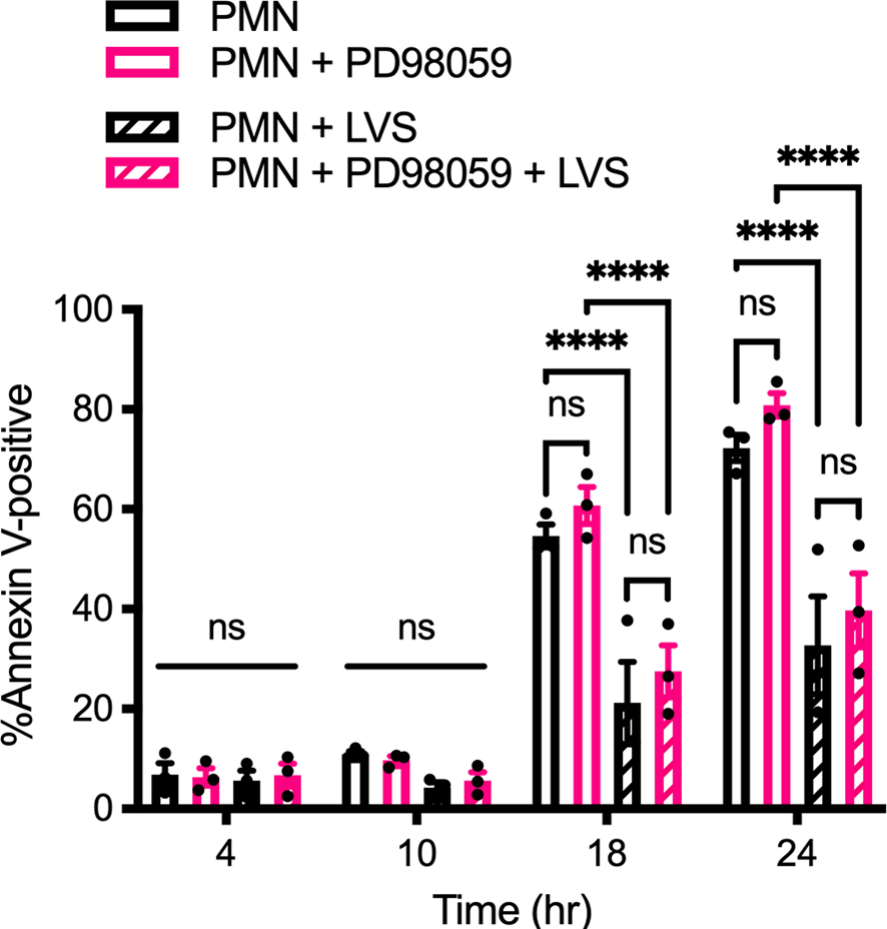
PD98059 does not alter the rate of apoptosis. Effects of the MEK/ERK inhibitor PD98059 on apoptosis of control and LVS-infected neutrophils was quantified using Annexin V-FITC/PI staining and flow cytometry at 4, 10, 18 and 24 hours. Data are the mean ± SEM of three independent experiments. ns, not significant. ****p<0.0001, as indicated.

In marked contrast to the data obtained for p38 MAPK, inhibition of MEK1/2 signaling using 50 μM PD98059 did not significantly alter the rate of PMN apoptosis in the presence or absence of LVS (**Figure 2**), and ERK phosphorylation was not increased above background at other time points examined (**Supplementary Figure 1B**). Taken together, these data demonstrate that both p38 MAPK and MEK/ERK signaling are activated in infected PMNs, but only p38 MAPK is required for *F. tularensis* to extend cell lifespan.

### PI3K Activity Is Required for Survival of Both Infected and Uninfected Neutrophils

Class I PI3Ks catalyze conversion of PI(4,5)P2 to PI(3,4,5)P3 and have been extensively studied (Hawkins et al., 2010; Fortin et al., 2011). Class IA PI3K isoforms (PI3Kα, PI3Kβ, and PI3Kδ) are important for growth and survival of many types of cells and in neutrophils and macrophages play additional roles in regulation of FcγR-mediated phagocytosis, cell adhesion, priming and NADPH oxidase activation. On the other hand, the lone Class IB PI3K isoform (PI3Kγ) is activated by heterotrimeric G-protein coupled receptors (GPCRs) and plays a specific role in regulating neutrophil migration to sites of infection and inflammation.

**Figure 3:**
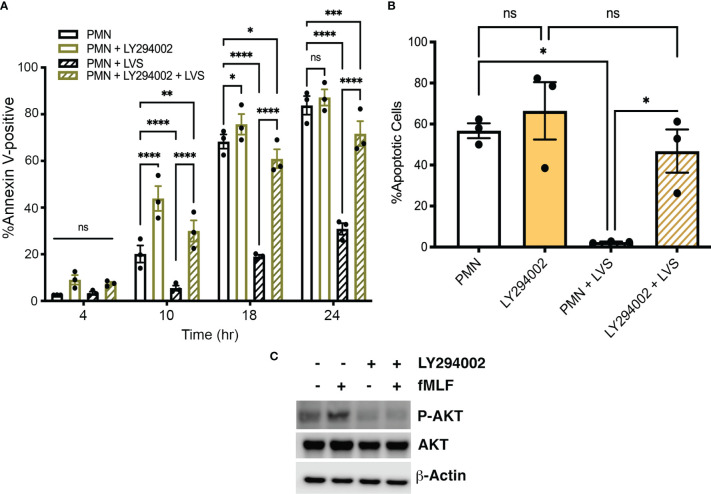
The pan-PI3K inhibitor LY294002 accelerates neutrophil death. **(A, B)** The effect of LY294002 on apoptosis of control and LVS-infected neutrophils was assessed at 4, 10, 18, and 24 hours by Annexin V-FITC/PI staining and flow cytometry **(A)** and at 18 hours by analysis of nuclear morphology **(B)**. Data are the mean ± SEM of three independent experiments. ns, not significant. *p<0.05, **p<0.01, ***p<0.001, ****p<0.0001, as indicated. **(C)** Immunoblots of cell lysates show that LY294002 prevents fMLF-triggered AKT phosphorylation. Total AKT is shown for comparison with β-actin as the loading control. Data shown are representative of three determinations.

LY294002 is a competitive, reversible pan-Class I PI3K inhibitor (Vanhaesebroeck et al., 1997; Juss et al., 2012) and we show here that pretreatment of neutrophils with this drug accelerated constitutive apoptosis of control, uninfected PMNs as indicated by Annexin V-FITC/PI staining at 10 and 18 hours (**Figure 3A**). Apoptosis of LVS-infected cell was also significantly accelerated by LY294002 as indicated by Annexin V-FITC/PI staining at 10, 18 and 24 hpi (**Figure 3A**) and analysis of nuclear condensation at 18 hpi (**Figure 3B**). Of note, the role of Class I PI3Ks in *Francisella* phagocytosis is opsonin-dependent, as uptake of complement-opsonized bacteria is PI3K-dependent whereas uptake of unopsonized bacteria is not (Clemens and Horwitz, 2007; Parsa et al., 2008). Importantly, we have shown that both opsonized and unopsonized LVS delay PMN death and we used unopsonized bacteria in this study to avoid possible confounding effects of LY294002 on infection efficiency (Schwartz et al., 2012; Kinkead et al., 2018).

AKT (also called protein kinase B) is a major Class I PI3K effector. Phosphorylation of AKT on S473 is commonly used as an indicator of PI3K activation and the formyl peptide fMLF is known to activate neutrophils in a PI3K-dependent manner (Wang et al., 2003). To demonstrate LY294002 efficacy, PMNs were stimulated with fMLF for 3 minutes in the presence and absence of this inhibitor. Immunoblotting of cell lysates demonstrated the ability of fMLF to trigger enhanced AKT phosphorylation in PMNs that was sensitive to inhibition by LY294002, whereas total AKT levels and the β-actin loading control were unchanged (**Figure 3C**). Based on these data, we conclude that PI3K activity is required for delayed apoptosis of *F. tularensis*-infected neutrophils and also regulates constitutive apoptosis of the uninfected controls.

### PI3Kα Plays a Specific Role in Extended Survival of *F. tularensis*-Infected Neutrophils

Our next objective was to determine if a specific Class IA PI3K isoform was required for longevity of *F. tularensis*-infected neutrophils. To this end, we took advantage of inhibitors that have been designed to target PI3K isoforms individually or in combination (**Table 1**). Specifically, we pretreated neutrophils with 10 μM HS-173, a specific PI3Kα inhibitor, 20 μM of the PI3Kα/PI3Kδ-selective inhibitor GDC-0941 (also called Pictilisib), 1 μM of the PI3Kδ-selective inhibitors IC-87114 and PIK-294, or 100 μM of the PI3Kβ-selective inhibitor TGX-221 (Juss et al., 2012; Sarker et al., 2015; Setti et al., 2016; Zillikens et al., 2021) and monitored apoptosis over the course of 24 hours in the presence and absence of LVS.

**Figure 4:**
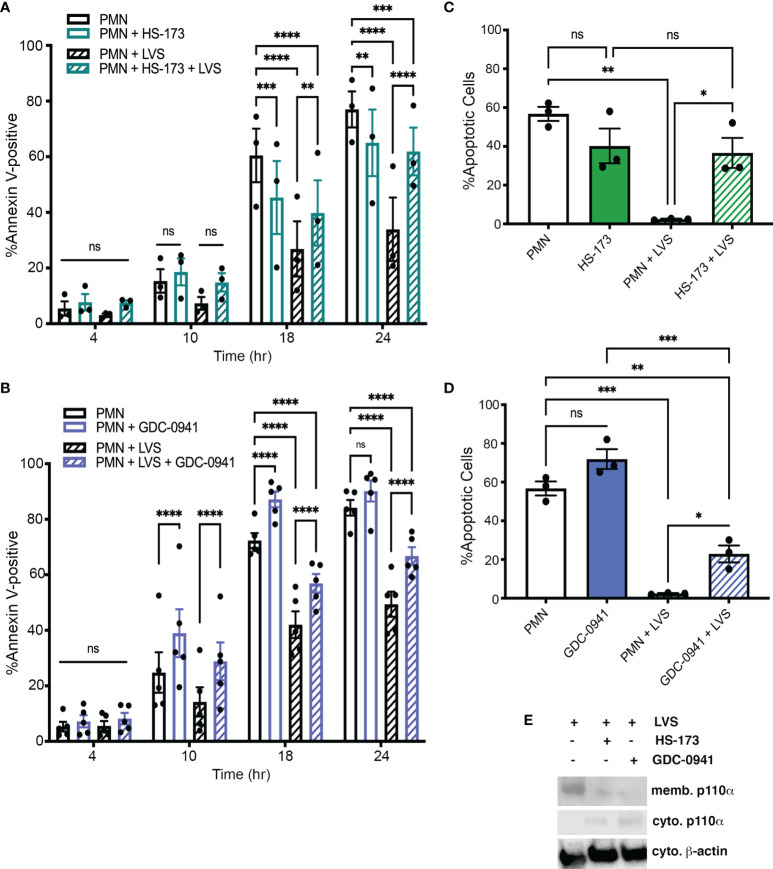
Class IA PI3Kα activity is required for survival after *F. tularensis* infection. PMNs were treated with HS-173 or GDC-0941 and then incubated for up to 24 hours in the presence and absence of LVS. **(A, B)** Apoptosis was assessed at 4, 10, 18, and 24 hours using Annexin V-FITC/PI staining and flow cytometry. Data are the mean ± SEM (n=3). ns, not significant. **p<0.01, ***p<0.001, ****p<0.0001, as indicated. **(C, D)** Percentage of neutrophils with apoptotic nuclei at 18 hours. Data are the mean ± SEM (n=3). ns, not significant. *p<0.05, **p<0.01, ***p<0.001, as indicated. **(E)** HS-173 and GDC-0941 impair LVS-induced membrane translocation of p110α as judged by immunoblotting of subcellular fractions. Representative of three determinations. Memb., membranes. Cyto, cytosol.

Our data demonstrate that HS-173 had no effect on apoptosis at 4 or 10 hours. Thereafter, apoptosis of the LVS-infected cells was markedly accelerated, whereas death of the uninfected control cells was significantly reduced (**Figure 4A**). On the other hand, treatment with the selective PI3Kα/PI3Kδ inhibitor GDC-0941 significantly increased apoptosis of both control and LVS-infected PMNs at 10, 18 and 24 hours (**Figure 4B**). The effect of each drug on cell viability was also assessed by analysis of nuclear morphology at 18 hours (**Figures 4C, D**), whereas efficacy was demonstrated by their ability to diminish LVS-stimulated membrane translocation of p110α, the catalytic subunit of PI3Kα (**Figure 4E**). Based on these data, we conclude that that the Class I PI3K isoforms required to prevent apoptosis of control and infected PMNs are distinct. Specifically, our data demonstrate that inhibition of PI3Kα is sufficient to trigger apoptosis after *F. tularensis* infection. In contrast, inhibition of both PI3Kα and PI3Kδ is required for accelerated apoptosis of the uninfected controls.

**Figure 5:**
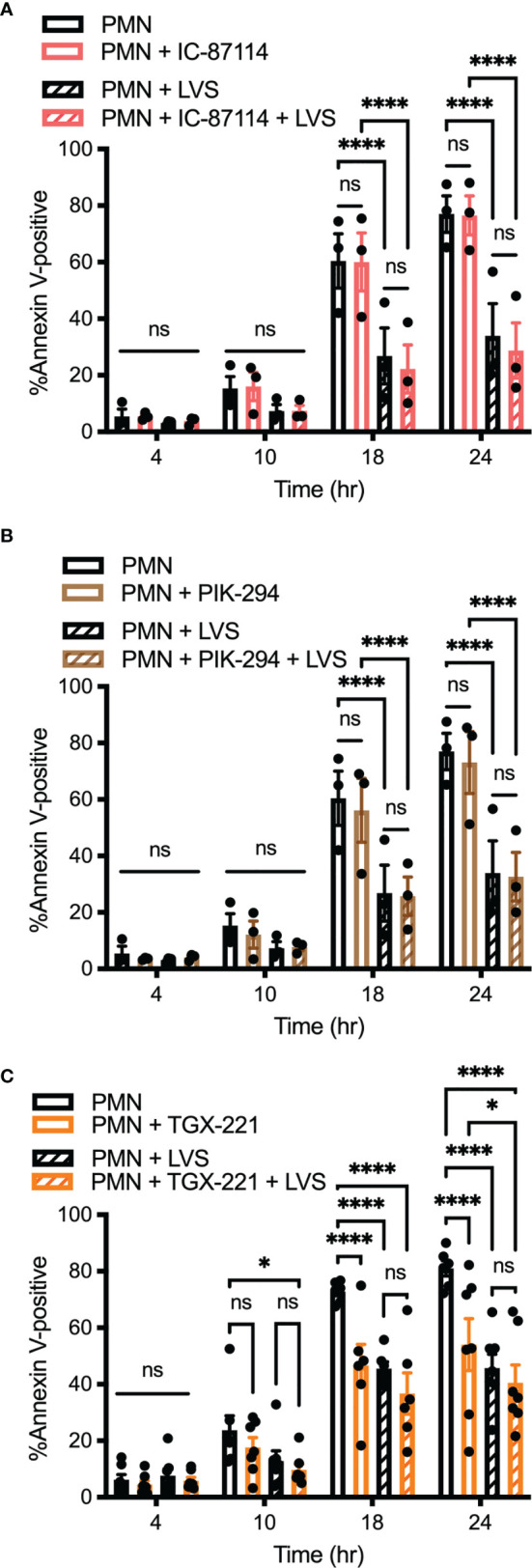
Inhibition of Class I PI3K isoforms PI3Kβ, PI3Kδ, and PI3Kγ does not prevent neutrophil apoptosis inhibition by *F. tularensis*. PMNs were left in medium alone or treated with IC-87114 **(A)** or PIK-294 **(B)** or TGX-221 **(C)** prior to further incubaction in the presence and absence of LVS. Apoptosis was quantified at 4, 10, 18, and 24 hours using Annexin V-FITC/PI staining and flow cytometry. Data are the mean ± SEM of three independent experiments. Ns, not significant. *p<0.05, ****p<0.0001, as indicated.

In another series of experiments, PMNs were treated with 1 μM IC-87114 or 1 μM PIK-294, both of which preferentially inhibit PI3Kδ but have some activity against PI3Kβ and PI3Kγ. Neither of these drugs altered the rate of apoptosis of control or LVS-infected PMNs (**Figures 5A, B**). In our final series of PI3K experiments we tested TGX-221, which preferentially inhibits PI3Kβ, but has some activity against PI3Kδ and PI3Kγ. We now show that 100 μM TGX-221 profoundly diminished apoptosis of uninfected PMNs at 18 and 24 hours, yet had no significant effect on LVS-infected cells at any of the time points examined (**Figure 5C**).

Collectively, our data suggest that Class I PI3Ks play complex roles in PMN survival and death. In particular, we identify PI3Kα activity as critical for survival of neutrophils infected with *F. tularensis* whereas inhibition of PI3Kβ enhanced survival of the uninfected controls. The data also indicate that simultaneous inhibition of PI3Kα and PI3Kδ, but not specific or selective inhibition of either isoform alone significantly accelerates PMN apoptosis in the absence of infection.

### AKT Activity Is Dispensable for Infected Neutrophil Survival

**Figure 6:**
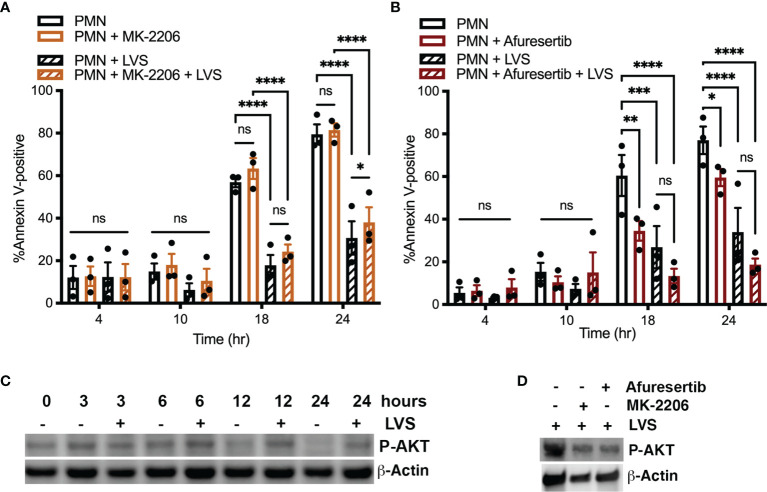
AKT activity is not required for neutrophil survival. **(A, B)** Neutrophils were treated with MK-2206 for 30 minutes **(A)** or Afuresertib for 60 minutes **(B)** prior to further incubation in the presence or absence of LVS. Apoptosis was assessed at 4, 10, 18, and 24 hours using Annexin V-FITC/PI staining and flow cytometry. Data are the mean ± SEM of three independent experiments. ns, not significant. *p<0.05, **p<0.01, ***p<0.001, ****p<0.001, as indicated. **(C, D)** Immunoblots of cell lysates were probed to detect phosphorylated AKT (P-AKT) with β-actin as the loading control. **(C)** Extent of AKT phosphorylation at 0, 3, 6, 12 and 24 hours in uninfected and infected cells, as indicated. **(D)** Effects of Afuresertib and MK-2206 on AKT phosphorylation in LVS-infected cells. Data shown are representative of three determinations.

As noted above, AKT is a commonly studied Class I PI3K effector, also known as protein kinase B. There are three isoforms of AKT, and AKT1 and AKT2 are expressed in neutrophils (Chen et al., 2010). To determine if AKT played a role in modulating neutrophil apoptosis, we treated cells with the allosteric pan-AKT inhibitor MK-2206 (Yamaji et al., 2017). In our hands, pretreatment of neutrophils 1 μM MK-2206 had no discernable effect on the rate of neutrophil apoptosis in the presence or absence of LVS as judged by flow cytometry (**Figure 6A**). As the effects of MK-2206 can be transient (Will et al., 2014; Johnson et al., 2021), we also tested the newer and more potent AKT inhibitor, Afuresertib (Yamaji et al., 2017). Consistent with the aforementioned data, pretreatment with 10 μM Afuresertib had no significant effect on the lifespan of neutrophils infected with LVS, but significantly reduced the rate of apoptosis of the uninfected cells at 18 and 24 hours (**Figure 6B**). These data were confirmed by analysis of nuclear morphology (data not shown).

Notably, our dot blot arrays did not detect enhanced AKT phosphorylation in resting or infected cells at the examined time points (**Figure 1A** and **Supplementary Figure 1B**). Further analysis of cell lysates using conventional immunoblotting suggest that AKT phosphorylation was maintained at a moderate level in LVS-infected cells over 24 hpi yet declined by 12 and 24 hours in the aged, uninfected controls (**Figure 6C**). Low level AKT phosphorylation in infected PMNs was independently confirmed using flow cytometry as was its diminished phosphorylation in the aged, uninfected controls (data not shown). As expected, AKT phosphorylation was sensitive to inhibition by MK-2206 and Afuresertib (**Figure 6D**). Considered together, these data suggest that AKT is not an essential regulator of neutrophil apoptosis during *F. tularensis* infection.

### *F. tularensis* Requires NF-κB Activation to Prolong Neutrophil Lifespan

**Figure 7:**
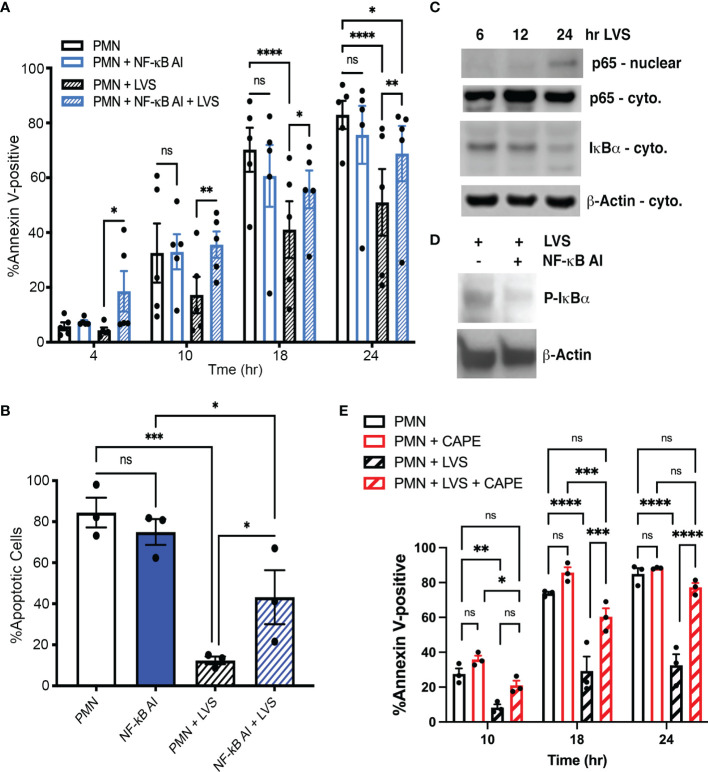
Inhibition of NF-κB selectively accelerates death of *F. tularensis*-infected neutrophils. **(A)** PMNs were pretreated with NF-κB AI for 30 minutes and then incubated for up to 24 hours in the presence and absence of LVS. Apoptosis was assessed at the indicated time points using Annexin V-FITC/PI staining and flow cytometry. Data are the mean ± SEM of five independent experiments. ns, not significant. **p<0.01, ***p<0.001, ****p<0.0001, as indicated. **(B)** Percentage of cells with apoptotic nuclei. Data are the mean ± SEM of three independent experinents. ns, not significant. *p<0.05, ***p<0.001, as indicated. **(C)** Immunoblots of subcellular fractions show p65 translocation into the nucleus and disappearance of IκBα from the cytosol of LVS-infected neutrophils. β-actin is the loading control. Data are representative of three determinations. **(D)** Immunoblots of cell lysates demonstrate the abilty of NF-κB AI to inhibit IκBα phosphorylation. β-actin is the loading control. Representative of three determinations. **(E)** PMNs were pretreated with CAPE and then incubated for up to 24 hours in the presence and absence of LVS. Apoptosis was assessed at the indicated time points as in panel **(A)**. Data are the mean ± SEM (n=3). ns, not significant. *p<0.05, **p<0.01, ***p<0.001, ****p<0.0001, as indicated.

NF-κB plays a key role in neutrophil survival during infection and inflammation by controlling expression of genes that encode important anti-apoptotic regulatory factors (Schwartz et al., 2013). We have shown that several NF-κB target genes are significantly differentially expressed during LVS infection, including *BCL2A1, BIRC3, BIRC4, TNFAIP3, CFLAR* and *CAST*, which encode A1, cIAP2, XIAP, A20, FLIP and calpastatin, respectively (Lawrence, 2009). Thus, we predicted that NF-κB activity may be essential in our system for infected cell survival. To test this hypothesis, we utilized NF-κB AI (NF-κB Activation Inhibitor) (Tsai et al., 2017). The data in **Figures 7A, B** indicate that NF-κB AI significantly accelerated apoptosis of LVS-infected PMNs at all time points examined. In sharp contrast, inhibition of NF-κB did not significantly alter the rate of apoptosis of uninfected PMNs, confirming published data (Dyugovskaya et al., 2011). Immunoblotting of subcellular fractions demonstrated LVS-induced NF-κB activation as indicated by nuclear translocation of the p65 subunit that was coupled to degradation of its inhibitor IκBα (**Figure 7C**). Proteolysis requires prior phosphorylation of IκBα and this step of the NF-κB activation cascade was inhibited by NF-kB AI, thereby demonstrating its efficacy (**Figure 7D**). Additionally, a role for NF-kB in infected PMN longevity was independently confirmed using CAPE (**Figure 7E**) (Armutcu et al., 2015). We therefore conclude that NF-κB is essential for delayed apoptosis of *F. tularensis*-infected neutrophils.

### Inhibitor Efficacy Is Not Attributable to Toxicity and Can Be Uncoupled From Phagocytosis

Data published by us and others demonstrate that *F. tularensis* rapidly releases bacterial lipoproteins (BLPs) and other unidentified factors that act at a distance to modulate PMN function and lifespan prior to binding and phagocytosis (Moreland et al., 2009; Allen, 2013; Kinkead et al., 2018). For this reason, we sought to determine if phagocytosis was essential for the prosurvival signaling identified in this study. To address this question, direct contact between neutrophils and bacteria was prevented by incubation on the opposite sides of 0.4 μm Transwell filters as previously described (Schwartz et al., 2012; Kinkead et al., 2018). Under these conditions, the p38 MAPK inhibitor SB203580 and the PI3K inhibitors LY294002, HS-173 and GDC-0941 retained their ability to significantly accelerate apoptosis, whereas the effects of NF-κB AI were diminished (*p*=0.09) (**Supplementary Figure 3**).

As it is established that viable *F. tularensis* prolongs neutrophil lifespan whereas killed bacteria do not (Schwartz et al., 2012), we performed additional experiments to determine if any of the drugs used in this study was toxic to LVS. To this end, each agent was added to BHI broth and bacterial growth was quantified over 18 hours at 37°C. By this assay, most drugs were without effect, but NF-κB AI was bacteriostatic (**Supplementary Figure 4** and data not shown). To address this limitation, we performed additional experiments using CAPE, a structurally distinct NF-κB inhibitor that has previously been shown not to effect *Francisella* growth in broth or its ability to be phagocytosed (Santic et al., 2010). The data in **Supplementary Figure 4** confirm that CAPE is not toxic and further support the notion that NF-κB is important for infected PMN longevity, as shown in **Figure 7**.

## Discussion

The ability of MAPKs, Class I PI3Ks and NF-κB to influence diverse neutrophil functions including migration, phagocytosis, oxidant production, cytokine secretion and lifespan is unequivocal. At the same time, a majority of studies have focused on responses to individual receptor ligands of host or microbial origin, such as GM-CSF or LPS. Much less is known about responses to whole microbes or other complex stimuli, and in most studies roles for enzyme isoforms were not interrogated. The results of this study demonstrate specific roles for p38 MAPK, PI3Kα and NF-κB in PMN apoptosis inhibition by *F. tularensis* and identify a distinct requirement for PI3Kα and PI3Kδ in regulation of human neutrophil lifespan in the absence of infection.

MEK/ERK signaling is activated in neutrophils in response to growth factors such as G-CSF and GM-CSF, LPS, hypoxia, or host inflammatory mediators such as IL-8, C5a, and LTB_4_ (Klein et al., 2000; Akgul et al., 2001; Klein et al., 2001; Geering and Simon, 2011). The central mechanisms of ERK-mediated apoptosis inhibition are phosphorylation of caspase-9, which curtails its activity, and phosphorylation of BAD at S112 which favors its sequestration in the cytosol, thereby helping to sustain OMM integrity (Allan et al., 2003; Perianayagam et al., 2004). We show here that although ERK2 phosphorylation was enhanced in neutrophils 10 hours after infection with *F. tularensis*, inhibition of ERK1/2 with PD90859 did not alter the rate of PMN apoptosis in the presence or absence of infection. This is likely due to redundant mechanisms of apoptosis inhibition in our system. For example, PIM2 can also phosphorylate BAD, and we have shown that *PIM2* expression is upregulated by LVS infection (Yan et al., 2003; Schwartz et al., 2013). In addition, MCL-1 and A1 are abundant in LVS-infected neutrophils and sustain mitochondrial integrity by binding and sequestering BAX and BAK, which are generally believed to play a greater role in disrupting neutrophil mitochondria than BAD (Schwartz et al., 2013; McCracken and Allen, 2014; McCracken et al., 2016). Finally, XIAP is an inhibitor of apoptosis protein that binds directly to caspase-9 and caspase-3 to block their catalytic activity and also inhibits processing of the respective proenzymes to their mature, active forms (Geering and Simon, 2011; Schwartz et al., 2013). XIAP is maintained at high levels in LVS-infected PMNs *via* increased expression of the *BIRC4* gene and by upregulation of calpastatin (*CAST*), which prevents calpain-mediated XIAP degradation in healthy cells and during LVS infection (Luo and Loison, 2008; Schwartz et al., 2013; McCracken and Allen, 2014; McCracken et al., 2016).

p38 MAPK is of interest as it can enhance, inhibit or have no effect on PMN apoptosis in a context-specific manner (Luo and Loison, 2008; Geering and Simon, 2011; McCracken and Allen, 2014). In neutrophils, the pro-death effects of p38 MAPK are commonly linked to phosphorylation of p47*^phox^*, NADPH oxidase activation and toxic oxidant production, whereas constitutive apoptosis of PMNs at rest is p38-independent (Frasch et al., 1998; Klein et al., 2001; Luo and Loison, 2008; Geering and Simon, 2011)(**Figure 1D**). In this regard it noteworthy that *F. tularensis* uses multiple strategies to disrupt NADPH oxidase assembly and activity in PMNs, and phosphorylation of p47*^phox^* is significantly diminished (McCaffrey et al., 2010; Kinkead and Allen, 2016). A role for p38 MAPK in prolonging PMN survival has been demonstrated in hypoxia, exposure to IL-32γ or dexamethasone and during infection with *Coxiella burnetii*, and we extended this list to include *F. tularensis* (Klein et al., 2001; Saffar et al., 2008; Dyugovskaya et al., 2011; Allaeys et al., 2014; Cherla et al., 2018).

At the molecular level, p38 favors survival by enhancing MCL-1 abundance, by direct phosphorylation and inhibition of caspase-3 and caspase-8 or by stimulating IL-8 secretion, and in many instances collaborates with PI3K or NF-κB (Alvarado-Kristensson et al., 2004; Saffar et al., 2008; Geering and Simon, 2011; Dyugovskaya et al., 2011; Cherla et al., 2018). Notably, p38 also has effects on metabolism, and like AKT can phosphorylate GSK3β to relieve inhibition of glycogen synthase (Rayasam et al., 2009). p38 stimulates glucose uptake *via* membrane translocation of GLUT1 and phosphorylates MK2 to stimulate PFKFB3 and amplify glycolysis at the level of PFK (Schuster et al., 2007; Novellasdemunt et al., 2013). Precisely how p38 contributes to apoptosis inhibition in our system remains to be determined. Live *F. tularensis* does not trigger secretion of IL-8, but effects on glucose metabolism are attractive, as expression of *SCL2A1*, which encodes GLUT1, and *PFKFB3* is enhanced (Schwartz et al., 2012; Schwartz et al., 2013).

A central finding of this study is our identification of distinct roles for Class IA PI3K isoforms in neutrophil survival in the presence and absence of *F. tularensis* infection. We identified a critical role for PI3Kα in delayed apoptosis of LVS-infected PMN whereas simultaneous inhibition of PI3Kα and PI3Kδ was required to undermine survival of the uninfected controls. Our findings support and extend published data which demonstrate that the roles of PI3K isoforms in cell survival are complex sometimes contradictory. Thus, although deactivation of PI(3,4,5) P_3_ signaling accompanies constitutive PMN apoptosis, simultaneous inhibition or deletion of at least three Class I PI3K isoforms is required to overcome the pro-survival effects of GM-CSF, and in keeping with this combined loss of PI3Kδ and PI3Kγ is not sufficient to alter resting PMN lifespan (Zhu et al., 2006; Juss et al., 2012). On the other hand, the ability of PI3Kβ to accelerate PMN apoptosis under certain circumstances may explain the increased longevity of TGX-221-treated cells shown in **Figure 5C**. Precisely what accounts for the survival-enhancing effect of HS-173 on uninfected PMNs is unknown, but this is negated and reversed by GDC-0941 (**Figure 4**). Although PI3Kδ is generally dispensable for PMN survival (Juss et al., 2012) (**Figures 5A, B**), a role for p38 and PI3Kδ in IL-8 and MIP-mediated apoptosis inhibition has been described (Fortin et al., 2011). Whether p38 MAPK is directly linked to PI3Kα and/or PI3Kδ in our system remains to be determined. In addition, we speculate that the survival advantage conferred by selective inhibition of PI3Kα in resting neutrophils might be explained by its relatively low abundance in this cell type, which may indirectly favor signaling by the other more abundant isoforms.

*PIK3CA*, which encodes the p110α catalytic subunit of PI3Kα, is mutated and overexpressed in many human cancers, and for this reason isoform-specific inhibitors have been developed as candidate therapeutics (Runman et al., 2016). HS-173 binds with high affinity to p110α and exhibits antitumor activity *in vivo* and *in vitro* (Landry et al., 2020). In cancer cell lines, HS-173 causes cell cycle arrest and apoptosis *via* the intrinsic pathway which has been linked to disruption of growth factor, insulin and TGFβ receptor signaling (Lee et al., 2013; Runman et al., 2016). Relevant downstream targets and pathways include AKT-dependent phosphorylation of BAD, caspase-9, and GSK3β in addition to effects on autophagy (Rane and Klein, 2009; Fan et al., 2017). Expression of *HIF1A* (HIF-1α) and *VEGFA* (VEGF) are also impaired. Notably, HIF-1α is important for neutrophil survival in normoxia as well as hypoxia, and *VEGFA* and many other HIF-1α target genes, but not *HIF1A* itself, are upregulated following LVS infection including *HK2, LDHA, PDK1* and *SCL2A1* (Cramer et al., 2003; Schwartz et al., 2013). Based on these data and evidence that p38 MAPK is also linked to GLUT1 and GSK3β, as noted above, we hypothesized that glycolysis and glucose metabolism may play a role in regulating PMN lifespan during *F. tularensis* infection, and this is supported by recently published data (Krysa and Allen, 2022). Other genes of interest that are differentially expressed in our system and have been linked to PI3K signaling include *GADD45B*, *SOD2*, *BNIP3* and *CDKN1A* (Schwartz et al., 2013; McCracken and Allen, 2014).

In addition to cell survival, PI3Kβ, PI3Kδ, and PI3Kγ have frequently been linked to PMN activation (Juss et al., 2012; Zillikens et al., 2021). PI3Kβ plays a specific role in Fc receptor phagocytosis and immune complex stimulation in collaboration with PI3Kδ. Accordingly, adhesion, spreading and ROS production are sensitive to inhibition by TGX-221 and IC-87114, but not HS-173. PI3Kγ is required for extravasation, chemotaxis and ROS production triggered by GPCR ligands. PI3Kδ has some influence on polarization and migration, but plays a broader role cytokine production and NADPH oxidase activation in PMNs following exposure to a wide range of stimuli of host and microbial origin including LPS, TNFα, IL-8, fMLF and C5a.

AKT is an extensively studied PI3K effector. The fact that AKT phosphorylation was not significantly induced by LVS despite the prominent role of PI3K in infected neutrophil survival was unexpected, as was the failure of AKT inhibitors to accelerate neutrophil death. However, this latter outcome is not without precedent, as PI3K-dependent/AKT-independent apoptosis inhibition has also been reported following treatment with IGF-1 and after infection by *L. major* (Himpe et al., 2008; Sarkar et al., 2013). Paradoxically, Afuresertib and other AKT inhibitors can also lead to PI3K activation, providing a potential explanation for the ability of Afuresertib to delay neutrophil death in our hands (Will et al., 2014). At the same time, redundancy with XIAP and p38 may negate any requirement for AKT with respect to inhibition of caspase-9, BAD or GSK3β (Datta et al., 1997; Takahashi et al., 1998; Dan et al., 2004; Gardai et al., 2004), as noted above.

The ability of NF-κB AI and CAPE to significantly accelerate apoptosis of *F. tularensis*-infected cells within four hours underscores the central role of the NF-κB pathway in neutrophil survival. In keeping with this, genes encoding NF-κB subunits p105 (*NFKB1*), p100 (*NFKB2*) and p65 (*RELA*) are upregulated by LVS as are the NF-κB target genes *IL1B, SOD2, GADD45B*, *CFLAR*, *TNFAIP3*, *BCL2A1*, *BIRC3*, and *BIRC4* that encode IL-1β, MnSOD, GADD45β, c-FLIP, A20, A1, cIAP2, and XIAP, respectively, and which act at various points to directly inhibit intrinsic and extrinsic apoptosis pathway activation, detoxify ROS, or promote survival (Schwartz et al., 2013). In particular, IL-1β is a PMN survival factor that can act in an autocrine and paracrine manner to stimulate NF-κB signaling (Schwartz et al., 2013). Moreover, GADD45β connects p38 MAPK to NF-κB; and MSK1/2, a downstream target of p38, phosphorylates NF-κB p65 leading to activation of this pathway (Hoffman and Liebermann, 2009; Schwartz et al., 2013; Christian et al., 2016). These data provide critical insight into how the expression of numerous distinct factors involved in cell survival are rapidly and concurrently altered, but precisely how NF-κB is linked to p38 MAPK and/or PI3K activation in *F. tularensis*-infected neutrophils remains to be determined.

**Figure 8:**
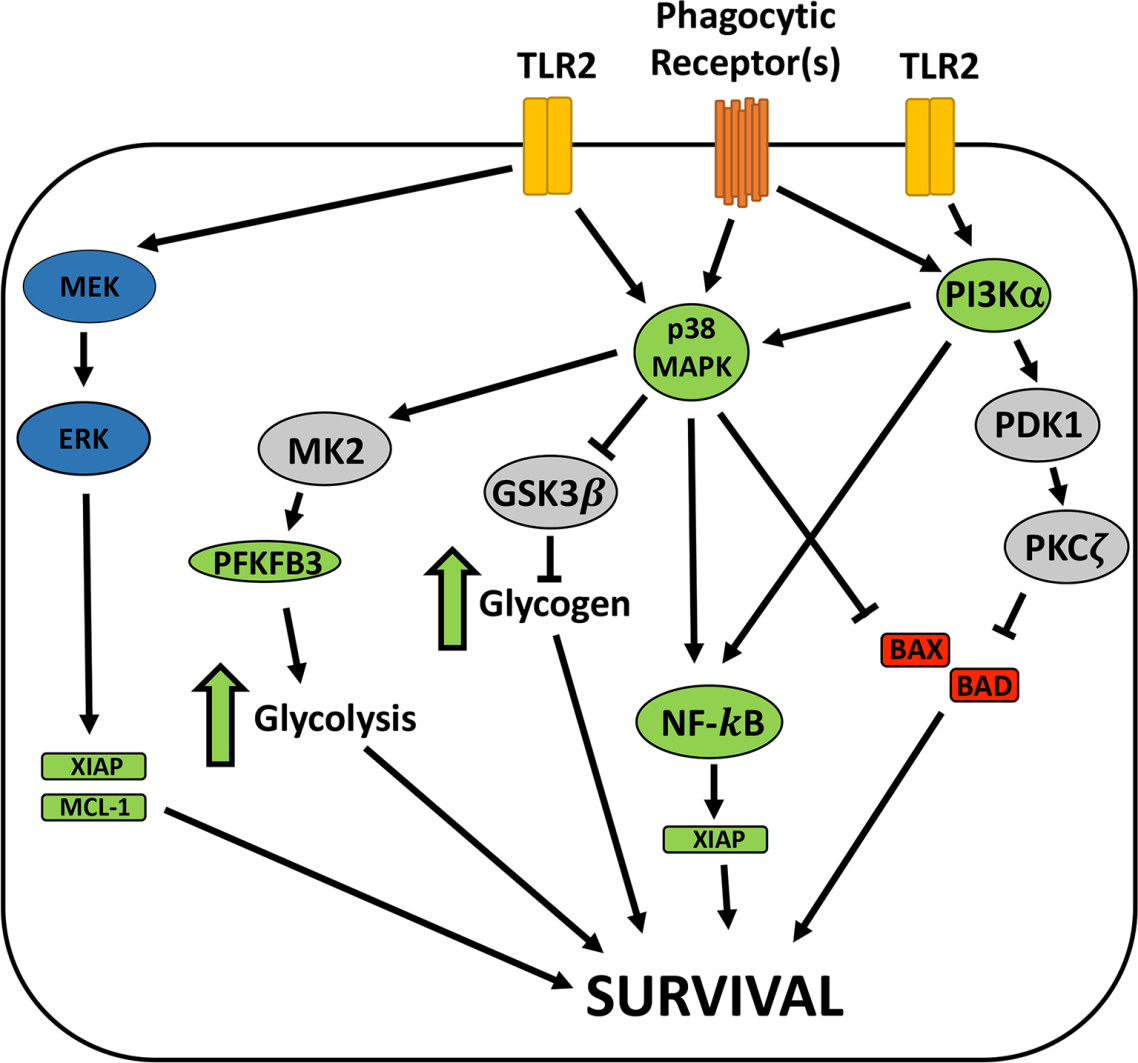
Model of *F. tularnesis* anti-apoptosis signaling in neutrophils. Signaling *via* TLR2 heterodimers and phagocytic receptors engage mutliple survival signaling pathways. MEK and ERK promote survival in some systems but not during *F. tularensis* infection. p38 MAPK and PI3Kα are required for extended neutrophil survival in our system and both may act *via* NF-κB, leading to expression of prosurvival factors, including XIAP. p38 MAPK may also be upstream of MK2 and GSK3β, and our recent data demonstrate that glycolysis and glycogen play key roles in neutrophil survival during *F. tularensis* infection and glycolysis pathway enzymes, including PFKFB3 are induced. At the same time, both PI3K and p38 are linked to inhibition of the proapoptosis factors BAD and BAX that disrupt mitochondrial integrity to initate the intrinsic appoptosis pathway. Having excluded a role for AKT, we speculate that other effectors of PI3K, such as PDK-1 and PKCζ may be involved, but this remains to be determined. Blue symbols, prosurvival enzymes with roles in other systems. Green symbols, molecular and pathways required for PMN survival during LVS infection. Red, proapoptosis factors. Grey, signaling intermediates not yet tested in our system.

Maximal inhibition of neutrophil apoptosis is achieved by the combined effects of intracellular live bacteria as well as bacterial lipoproteins (BLPs) and other factors that are secreted/released by extracellular *F. tularensis* prior to phagocytosis (Allen, 2013; Kinkead et al., 2018). *F. tularensis* PAMPs are detected by TLR2/1 and TLR2/6 but not TLR4, and a specific role for TLR2/1 in BLP-mediated survival signaling has been described (Kinkead and Allen, 2016; Kinkead et al., 2018). In contrast, *F. tularensis* LPS has an atypical structure and is not a TLR4 agonist, and LPS and the O-antigen capsule do not modulate PMN lifespan (Schwartz et al., 2012; Kinkead et al., 2018). Based on studies of whole blood, TLR2/1 survival signaling has been linked to PI3K, AKT and NF-κB and is associated with increases in A1 and MCL-1 as well as phosphorylation of BAD. In this case, PI3K is upstream of p38 and ERK, and apoptosis inhibition is sensitive to both PI3K and NF-κB inhibitors (Francois et al., 2005), and many of these players are relevant in our direct infection model. At the same time, the fact that conditioned medium, isolated BLPs and separation of bacteria and neutrophils in Transwells is less efficient at delaying apoptosis than direct infection indicates a role for intracellular bacteria in sustaining neutrophil viability (Schwartz et al., 2013; Kinkead et al., 2018) (**Supplementary Figure 3**). In this regard it is of interest that *F. tularensis* resides in a phagosome for four hours before entering the cytosol where replication ensues, and bacteria that persist in the phagosome but are defective for escape to the cytosol potently extend cell lifespan (McCaffrey and Allen, 2006; Schwartz et al., 2012; Kinkead and Allen, 2016). We do not yet know if phagosomal and cytosolic bacteria play distinct roles in apoptosis modulation. Thus, additional studies are needed to elucidate the roles of individual PMN receptors and bacterial localization on p38, PI3Kα and NF-κB and their shared and distinct downstream targets. A working model based on the results of this study is shown in **Figure 8**.

In summary, the results of this study provide fundamental insight into the role pro-survival signaling pathways in delayed apoptosis of human neutrophils infected with *F. tularensis*. We identified p38 MAPK, PI3K and NF-κB as essential yet excluded roles for ERK and AKT, results that distinguish *F. tularensis* from other stimuli described to date. In identifying critical roles for PI3Kα and PI3Kδ in resting PMN survival and PI3Kα in longevity of cells infected with *F. tularensis*, our data also fundamentally advance insight into the biology of Class IA PI3K isoforms in this cell type. Overall, our findings underscore the complexity of the signaling pathways that regulate PMN life and death.

## Data Availability Statement

The raw data supporting the conclusions of this article will be made available by the authors, without undue reservation.

## Ethics Statement

Studies involving human participants were reviewed and approved by the Institutional Review Boards of the University of Iowa and the University of Missouri and all participants provided written informed consent to participate in this study.

## Author Contributions

LK and SK designed and performed experiments, interpreted data and co-wrote the manuscript. L-AA conceived of the study, designed and performed experiments, interpreted data and co-wrote the manuscript. All authors contributed to the article and approved the submitted version.

## Funding

This study was supported by VA Merit Review Grant 1I01BX002108 and NIH/NIAID U54AI057160 and R01AI073835 funds awarded to L-AA. LK was supported in part by a predoctoral fellowship *via* NIH/NIAID T32AI007511. SK was supported in part by a University of Iowa post-comprehensive exam fellowship.

## Conflict of Interest

The authors declare that the research was conducted in the absence of any commercial or financial relationships that could be construed as a potential conflict of interest.

## Publisher’s Note

All claims expressed in this article are solely those of the authors and do not necessarily represent those of their affiliated organizations, or those of the publisher, the editors and the reviewers. Any product that may be evaluated in this article, or claim that may be made by its manufacturer, is not guaranteed or endorsed by the publisher.
