# Supplementary material for: Neutrophil Survival Signaling During Francisella tularensis Infection
Source: Front Cell Infect Microbiol. 2022 Jul 6;12:889290. doi: 10.3389/fcimb.2022.889290 (PMC9299441; doi:10.3389/fcimb.2022.889290)
Supplement: Supplementary file 1 [file DataSheet_1.pdf]

Supplementary Figures for Kinkead et al.

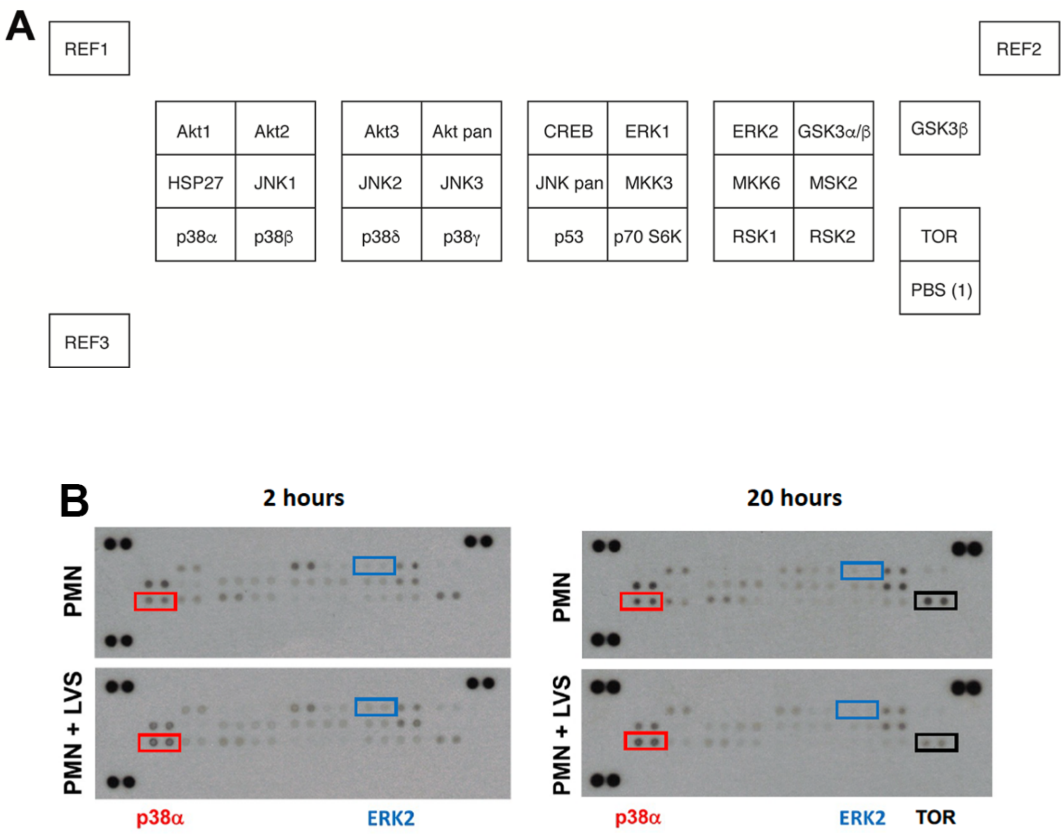

Supplementary Figure 1. Phospho-MAPK Dot Blots. (A) Spot map for R&D Systems human phospho-MAPK dot blot slide arrays. Each box contains two spots. Corner spots are positive (REF1-3) and negative (lower right) controls. **(B)** Dot blots were probed with lysates of control and infected cells prepared at 2 and 20 hours, as indicated. LVS infection did not enhance phosphorylation of p38α or ERK2 at these time points. TOR phosphorylation appeared slightly more abundant in uninfected cells at 20 hour. Representative of three determinations.

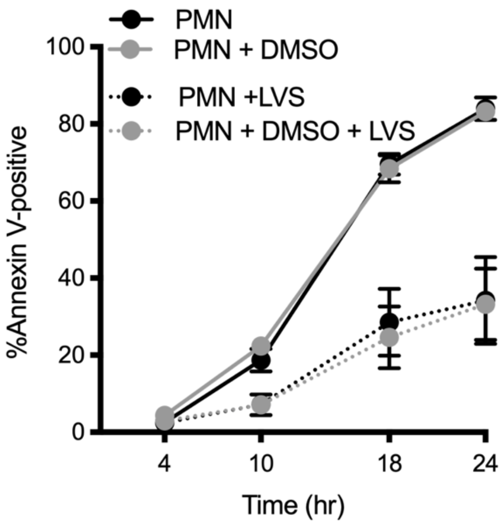

Supplementary Figure 2. DMSO does not alter the kinetics of neutrophil apoptosis. Control and LVS-infected neutrophils were incubated in medium with and without DMSO as the diluent control for the indicated amounts of time prior to analysis by Annexin V-FITC/Propidium Iodide co-staining and flow cytometry. Data show the percentage of Annexin V-FITC-positive cells at each time point and are the mean ± standard error of the mean (n=3). Where not visible, error bars are smaller than symbols.

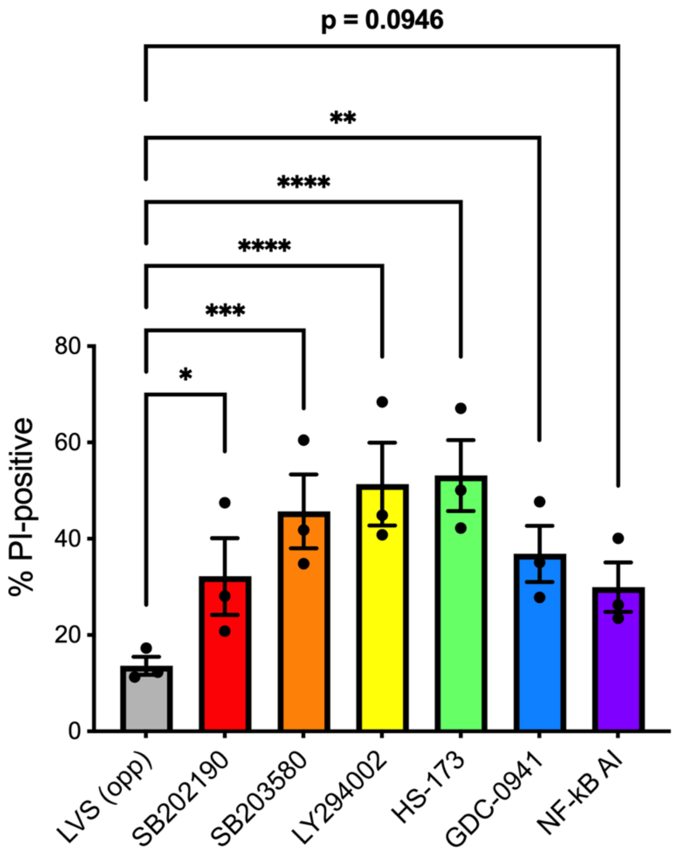

Supplementary Figure 3. Effects of signaling inhibitors on neutrophil viability in Transwells. Neutrophils and bacteria were added to the bottom and top chambers of Transwells containing a 0.4 µm pore filter supports, respectively. Inhibitors or medium alone were added, as indicated. After 24 hours at 37°C, neutrophils were collected, stained with propidium iodide (PI) and analyzed by flow cytometry. The percentage of PI-positive cells is shown as the mean ± standard error of the mean (n=3). *p<0.05, **p<0.01, ***p<0.001, as indicated. 'Opp' indicates that LVS were incubated on the opposite side of the Transwell from the neutrophils in the absence of inhibitors.

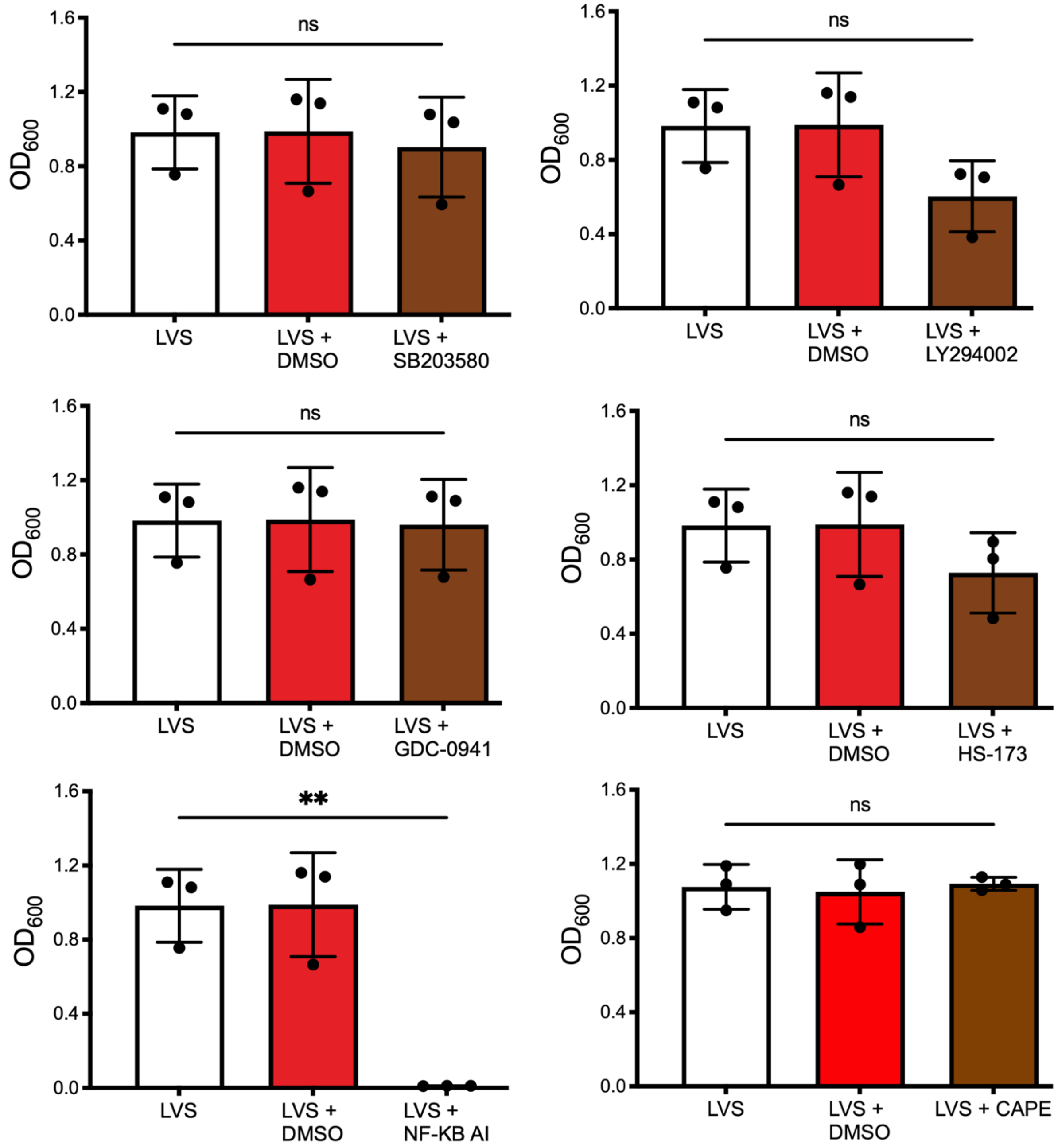

Supplementary Figure 4. Effects of signaling inhibitors on bacterial growth in broth. *F. tularensis* LVS was grown overnight at 37ºC with shaking at 200 rpm in brain heart infusion broth in the absence or presence of SB203580, LY294002, GDC-0941, HS-173, NF-κB AI, CAPE or the DMSO diluent, as indicated. Growth was monitored by measurement of the OD_{600}. Data for the 16 hour time point are shown as the mean ± standard error of the mean (n=3). ns, not significant. $^{**}p<0.01$.
